# Effects of leucine-enriched essential amino acid and whey protein bolus dosing upon skeletal muscle protein synthesis at rest and after exercise in older women

**DOI:** 10.1016/j.clnu.2017.09.008

**Published:** 2018-12

**Authors:** Daniel J. Wilkinson, Syed S.I. Bukhari, Bethan E. Phillips, Marie C. Limb, Jessica Cegielski, Matthew S. Brook, Debbie Rankin, William K. Mitchell, Hisamine Kobayashi, John P. Williams, Jonathan Lund, Paul L. Greenhaff, Kenneth Smith, Philip J. Atherton

**Affiliations:** a MRC/ARUK Centre of Excellence for Musculoskeletal Ageing Research, National Institute for Health Research Nottingham Biomedical Research Centre, University of Nottingham, Derby DE22 3DT, UK; b Ajinomoto Co., Inc., Tokyo 104-8315, Japan

**Keywords:** Muscle protein synthesis, Leucine, Ageing, Low dose amino acid supplementation, Exercise, Human metabolism, MPS, muscle protein synthesis, EAA, essential amino acids, WP, whey protein, LEAA, leucine enriched essential amino acids, LBF, leg blood flow, MBF, microvascular blood flow, BMI, body mass index, SMI, skeletal muscle index, 1-RM, 1 repetition maximum, PVDF, polyvinylidene difluoride, TBST, tris buffered saline/Tween 20, CEUS, contrast enhanced ultrasound

## Abstract

**Background & aims:**

Impaired anabolic responses to nutrition and exercise contribute to loss of skeletal muscle mass with ageing (sarcopenia). Here, we tested responses of muscle protein synthesis (MPS), in the under represented group of older women, to leucine-enriched essential amino acids (EAA) in comparison to a large bolus of whey protein (WP).

**Methods:**

Twenty-four older women (65 ± 1 y) received (*N* = 8/group) 1.5 g leucine-enriched EAA supplements (LEAA_1.5), 6 g LEAA (LEAA_6) in comparison to 40 g WP. A primed constant I.V infusion of ^13^C_6_-phenylalanine was used to determine MPS at baseline and in response to feeding (FED) and feeding-plus-exercise (FED-EX; 6 × 8 unilateral leg extensions; 75%1-RM). We quantified plasma insulin/AA concentrations, leg femoral blood flow (LBF)/muscle microvascular blood flow (MBF), and anabolic signalling via immunoblotting.

**Results:**

Plasma insulineamia and EAAemia were greater and more prolonged with WP than LEAA, although LEAA_6 peaked at similar levels to WP. Neither LEAA or WP modified LBF or MBF. FED increased MPS similarly in the LEAA_1.5, LEAA_6 and WP (*P* < 0.05) groups over 0–2 h, with MPS significantly higher than basal in the LEAA_6 and WP groups only over 0–4 h. However, FED-EX increased MPS similarly across all the groups from 0 to 4 h (*P* < 0.05). Only p-p70S6K1 increased with WP at 2 h in FED (*P* < 0.05), and at 2/4 h in FED-EX (*P* < 0.05).

**Conclusions:**

In conclusion, LEAA_1.5, despite only providing 0.6 g of leucine, robustly (perhaps maximally) stimulated MPS, with negligible trophic advantage of greater doses of LEAA or even to 40 g WP. Highlighting that composition of EAA, in particular the presence of leucine rather than amount is most crucial for anabolism.

## 1 Introduction

Healthcare for ageing populations represents a major socio-economic challenge, e.g. approximately 7% of the EU's GDP is currently used to provide healthcare for older people. With the ageing population set to continue to increase in coming years, it is predicted this figure will increase to more than 8% by 2060 [1]. Of paramount clinical concern is age associated muscle loss, termed sarcopenia, which occurs at a rate of ∼1%/y in men and ∼0.5%/y in women beyond the age of 50 y [2]. Such significant loss of muscle mass is an influential factor in age-related functional impairment, poor quality-of-life, the onset of metabolic/cardiovascular disease and an increased mortality risk [3], [4], [5].

Both physical activity and nutrition appear to be key regulatory factors in the control of muscle mass [6]. It is well established that following the intake of protein/amino acids there is a transient increase in MPS (peaking at ∼2 h; [7], [8]), which assists to replenish protein lost during periods of fasting and hence maintaining stable muscle mass in young adults. This “anabolic window” for increasing MPS with nutrition is extended when nutrition is combined with exercise [9], [10], [11]. Importantly, responsiveness to anabolic stimuli has been found to be significantly reduced in older adults. For example, postprandial rises in MPS in older adults are attenuated in comparison to young adults [12], [13], [14], [15], with reductions as great as 40% in those consuming the same amount of EAA [13]. Indeed, a recent study suggests older adults may require a greater quantity (∼0.4 g/kg vs. ∼0.24 g/kg in young) of nutrition to exact equivalent anabolic responses to that of young adults [16], [17]. The same anabolic blunting is also observed in response to acute [18], [19] and chronic resistance exercise training [20], [21].

The EAA, in particular leucine, have key roles in regulating the protein synthetic machinery [22], [23], [24], [25], [26]. Indeed 3 g of leucine alone in the absence of other AA can stimulate MPS to near maximal levels (equivalent to that observed from a large bolus of whey protein) in young adult males [24]. With this in mind it has recently been hypothesized that lower doses of EAA enriched with leucine may provide a robust and less satiating (or caloric) anabolic alternative to the larger quantities of protein that are suggested to be needed to be consumed in older age to maximize MPS [27], [28]. In support of this hypothesis we recently showed that despite providing more EAA substrate, a single bolus of 20 g WP provides no additional anabolic benefit to that provided by a low dose (3 g), low calorie, and leucine (40%) enriched EAA supplement (Amino L40, Ajinomoto) [27]. Studies such as this in older women are important since sexual dimorphism in protein metabolism has been shown in older age [14], [29] and most researchers study only older male populations. Therefore, as an extension to our previous studies, we quantified the effects of 1.5 g or 6 g leucine enriched (LEAA) EAA [40% LEU] nutrition (note: these doses are 50% and 200% of the doses that we previously tested [27]) in comparison to 40 g WP (a dose elicting an upper ceiling effect on MPS in younger males [30]) under rested and exercised conditions in muscles of older aged women.

## 2 Materials and methods

### 2.1 Subject characteristics and ethics

All studies were performed according to the Declaration of Helsinki and registered at clinicaltrials.gov (registration no. NCT02053441). Following ethical approval granted by the University of Nottingham Medical School Ethics Committee, twenty-four older post-menopausal women (8 in each group) matched for age and BMI (see Table 1 for subject demographics) were recruited from the local Derbyshire area via advertisement through the mail. To minimize confounding variables, exclusion criteria included: impaired mobility, history of diabetes, cardiovascular, pulmonary, liver or kidney disorders, those on contraindicated medications (NSAIDS, acetaminophen, HRT) and those currently undergoing active cancer therapies. Following recruitment and before inclusion in the project all volunteers were additionally screened by a physician (at least one week prior to the study day) by means of a medical questionnaire, physical examination and resting ECG, to exclude for any metabolic, respiratory, cardiovascular/vascular or claudication related disorders or other symptoms of ill health. All volunteers had normal blood chemistry, were normotensive (BP < 140/90), performed activities of daily living and recreation but were not involved in any formal exercise regimes, and were not on a weight controlled diet. During screening knee extensor strength was assessed via 1-RM, using the volunteer's dominant leg (Technogym, Gambettola, Italy). In addition, lean body mass was assessed via Dual X-ray Absorptiometry (DXA), and used to measure appendicular muscle mass and calculate skeletal muscle index (SMI) according to the following equation [31]:

Skeletal Muscle Index(SMI)=(Total Body Skeletal Muscle Mass/Total Body Mass)×100

**Table 1:** Subject demographics for each intervention group. Values are presented as mean ± SEM.

	LEAA_1.5	LEAA_6	WP
Age	65 ± 1 y	63 ± 1 y	66 ± 1 y
Height (m)	1.62 ± 0.02	1.64 ± 0.02	1.60 ± 0.02
Weight (kg)	65.4 ± 3.5	68.4 ± 5.4	65.0 ± 4.6
BMI (kg m^−2^)	25.1 ± 1.3	25.1 ± 1.3	25.0 ± 1.5
SMI	6.32 ± 0.11	6.18 ± 0.13	6.13 ± 0.35

All subjects provided written, informed consent to participate after all procedures and risks were explained. Following screening volunteers were randomly assigned, to one of three groups receiving either; 1) 40 g Whey Protein (WP, *N* = 8), 2) 1.5 g Leucine enriched essential amino acids (LEAA_1.5, *N* = 8) or 3) 6 g LEAA (LEAA_6, *N* = 8). Volunteers were requested not to modify their normal diet or activity regime before the study day and to refrain from any form of strenuous physical activity for 48 h prior to the acute study visit.

### 2.2 Study procedures

**Fig. 1:**
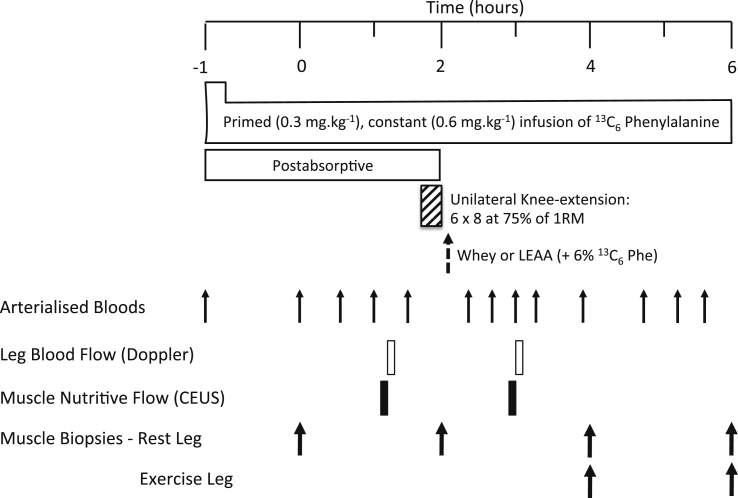
Study protocol: effects of leucine-enriched amino acids (LEAA) and whey protein (WP) at rest and after resistance exercise (RE) in older women. 1-RM, 1-repetition maximum; CEUS, contrast-enhanced ultrasound.

Study procedures followed our standard protocols as previosuly described by Bukhari et al., [27]. On the morning of the study (0800 h) following an overnight fast from 2000 h the previous day, volunteers had a cannula inserted into the antecubital vein of one arm for the infusion of a l-[ring-^13^C_6_]-phenylalanine tracer (Isotec, Sigma Aldrich; prime: 0.3 mg kg^−1^, constant: 0.6 mg kg^−1^ h^−1^), with a retrograde cannula inserted to sample arterialized blood from the dorsal capillary bed of the hand. Muscle biopsies were taken 1 h and 3 h after commencement of tracer infusion to permit assessment of basal (post-absorptive) MPS. Volunteers then performed a bout of unilateral knee extension resistance exercise previously shown [18] to maximally stimulate MPS (6 × 8 repetitions at 75% of their pre-determined 1-RM using the volunteer's dominant leg with 2 min inter-set rest period). If the volunteer failed to complete 8-repetitions, then an inter-set break was allowed before moving on to the next set. This happened regularly with sets 5 & 6. Immediately following the exercise each volunteer consumed their assigned supplement, either 40 g of whey protein (WP), 1.5 g (LEAA_1.5) or 6 g (LEAA_6) of LEAA (“Amino L40”; Ajinomoto Inc.,) prepared in water (250 ml). The AA composition of each supplement is shown in Table 2. This unilateral study design meant that the non-exercised leg was exposed to the effect of feeding alone (‘FED’), while the exercised leg was exposed to the combination of feeding and exercise (‘FED-EX’). Further muscle biopsies were then taken 2 and 4 h after feeding to permit assessment of MPS over the intervening periods. Muscle biopsies were collected from m. *vastus lateralis* using the conchotome technique [32] after induction of local anaesthesia via infiltration of 5 ml 1% lignocaine, washed with ice-cold phosphate buffered saline before being snap frozen in liquid N_2_ and stored at −80 °C until analysis. Blood samples and blood flow/vascular measurements were collected as outlined in the study schematic (Fig. 1).

**Table 2:** Essential amino acid composition of each type of feed (inclusive of data from Bukhari et al. [27]) Note: l-Lysine monohydrochloride and l-Histidine monohydrochloride monohydrate were used as l-Lysine and l-Histidine respectively in LEAA.

	LEAA (1.5 g), g	LEAA (3 g), g	LEAA (6 g), g	WP (20 g), g	WP (40 g), g
l-Leucine	0.6	1.2	2.4	2	4
l-Isoleucine	0.16	0.32	0.64	1.4	2.8
l-Valine	0.165	0.33	0.66	1.2	2.4
l-Threonine	0.14	0.28	0.56	1.4	2.8
l-Lysine	0.25	0.5	1.0	1.8	3.6
l-Methionine	0.05	0.1	0.2	0.4	0.8
l-Histidine	0.025	0.05	0.1	0.4	0.8
l-Phenylalanine	0.1	0.2	0.4	0.6	1.2
l-Tryptophan	0.01	0.02	0.04	0.4	0.8

#### 2.2.1 Measurement of plasma insulin and AA concentrations

Plasma insulin concentration was measured using a high-sensitivity human insulin enzyme-linked immunosorbent (ELISA) assay (DRG Instruments GmbH, Marburg, Germany). For AA analyses, 10 μl of a mix of stable isotopically labelled internal standards were added to 100 μl of plasma, treated with urease and deproteinised with 1 ml ice cold ethanol on ice for 20 min. Following centrifugation (17,000 × g; 10 min), the supernatant was decanted and evaporated to dryness under nitrogen. Following resuspension in 0.5 M HCl, lipids were extracted with 2 ml of ethyl acetate before the lower HCl phase was evaporated and AA were derivatized to their tBDMS esters. AA concentrations were quantified against a standard curve of known concentrations using GC–MS (Trace 1300-ISQ, Thermo Scientific, Hemel Hempstead, UK).

#### 2.2.2 Measurement of leg blood flow (LBF) and muscle microvascular blood flow (MBF)

Leg blood flow (LBF) was measured using Doppler ultrasound, with a 9-3 mHz probe positioned over the origin of the common femoral artery, and estimated as the product of vessel cross-sectional area and mean velocity over six cardiac cycles. Microvascular blood flow was measured using contrast enhanced ultrasound (CEUS) as previously described [33]. Briefly, Sonovue™ microbubbles (Bracco, Milan, Italy) were infused via an antecubital vein. An iU22 ultrasound scanner with 40 mm linear 9-3 mHz probes (Phillips Healthcare, Reigate, United Kingdom) secured on both anterior thighs was used to register micro-bubble appearance within the quadriceps muscle at rest and in response to FED and FED-EX (legs measured individually). Intermittent high mechanical index (MI) “flashes” disrupted micro-bubbles whilst continuous low MI recording measured the rate of micro-bubble replenishment. Infusion was at 2  ml min^−1^ for 1 min and 1  ml min^−1^ for 3 subsequent min. During the last 90 s of this measurement protocol, 3 × 30 s flash/replenishment recordings were made. Off line region-of-interest analysis using Q-Lab software (Phillips Healthcare, Surrey UK) was used to measure the plateau (“*A*” value) linear acoustic intensity and also the rate constant (“*β*” value); the product of which is proportional to volume blood volume tissue^−1^ s^−1^ and thus acts as a measure of muscle tissue microvascular blood flow. In total, 2 studies were done per participant: first 50–60 min before the exercise and second 50–60 min after the exercise session (Fig. 1).

#### 2.2.3 Measurement of MPS

Myofibrillar proteins were isolated, hydrolyzed and derivatized using our standard techniques [24]. Briefly, ∼25 mg of muscle biopsy tissue was homogenized in ice-cold homogenization buffer (50 mM Tris–HCl (pH 7.4), 50 mM NaF, 10 mM β-glycerophosphate disodium salt, 1 mM EDTA, 1 mM EGTA, 1 mM activated Na_3_VO_4_ (all Sigma–Aldrich, Poole, UK)) and a complete protease inhibitor cocktail tablet (Roche, West Sussex, UK) at 10 μl μg^−1^ of tissue. Following centrifugation at 13,000 × g for 5 min at 4 °C, the resulting insoluble pellet was washed three times with homogenization buffer to remove excess free AA and solubilized in 0.3 M NaOH to aid separation of the soluble myofibrillar fraction from the insoluble collagen fraction by subsequent centrifugation. The soluble myofibrillar fraction was precipitated using 1 M perchloric acid (PCA), pelleted by centrifugation and washed twice with 70% ethanol. The protein-bound AA were released by acid hydrolysis using 0.1 M HCl and 1 ml of Dowex ion-exchange resin (50W-X8-200) overnight at 110 °C. The free AA were purified, derivatized and the fractional synthesis rates (FSR) of the myofibrillar proteins was calculated using the precursor-product equation below:

$$\text{FSR}\;\left(\%/\text{h}\right)=\left[\frac{\Delta E_{\text{m}}}{E_{\text{p}}\cdot t}\right]\times 100$$

where Δ*E*_m_ is the change in enrichment of bound l-[*ring*-^13^C_6_] phenylalanine in two sequential biopsies, *t* is the time interval between two biopsies in hours, and *E*_p_ is the mean free l-[ring-^13^C_6_] phenylalanine enrichment in the intramuscular pool.

#### 2.2.4 Immunoblotting for AKT-mTORc1 signalling pathway activity (i.e. phosphorylation)

Immunoblotting was performed as previously described [26] using the sarcoplasmic fraction isolated from the MPS preparation. Sarcoplasmic protein concentrations were analysed using a NanoDrop ND1000 spectrophotometer (NanoDrop Technologies, Inc., Wilmington, DE-US) and sample concentrations adjusted to 2 μl μg^−1^ in 3 × Laemmli buffer to ensure equivalent protein loading onto pre-cast 12% Bis-Tris Criterion XT gels (BioRad, Hemel Hempstead, UK) of 20 μg/lane. Samples were separated electrophoretically at 200 V for 1 h, followed by wet transfer of proteins to PVDF membrane at 100 V for 45 min and subsequently blocking in 2.5% non-fat milk in 1 × Tris buffered saline/Tween 20 (TBST) for 1 h. Membranes were incubated in primary antibodies (1:2000 dilution in 2.5% BSA in TBS-T) overnight at 4 °C; p70S6K1^Thr389,^ 4EBP1^Ser65/70^, eEF2^Thr56^ (New England Biolabs, Hertfordshire, UK). Membranes were subsequently washed and incubated in HRP-conjugated secondary antibody (New England Biolabs, Hertfordshire, UK; 1:2000 in 2.5% BSA in TBST) at ambient temperature for 1 h, before being exposed to Chemiluminescent HRP Substrate (Millipore Corporation, Billerica, MA-US) for 5 min and bands quantified by Chemidoc XRS (BioRad, Hertfordshire, UK). All signals were within the linear range of detection; loading anomalies were corrected to coomassie [34].

### 2.3 Statistical analyses

Data are presented as means ± SEM, except if indicated. Data were checked for normal distribution using a Kolmogorov–Smirnov Test. Where the assumption of normality was not met, data were log transformed before further analyses. MPS data were analysed using a two-way ANOVA (feed type × time) with a Tukey's Post Hoc Test. Plasma and Immunoblotting data were analysed using two-way repeated measures ANOVA (feed type × time) with a Bonferroni correction. All data analysis was performed using GraphPad Prism (GraphPad Software Inc, San Diego, CA); the alpha level of significance was set at *P* < 0.05.

## 3 Results

### 3.1 Plasma AA and insulin concentrations

**Fig. 2:**
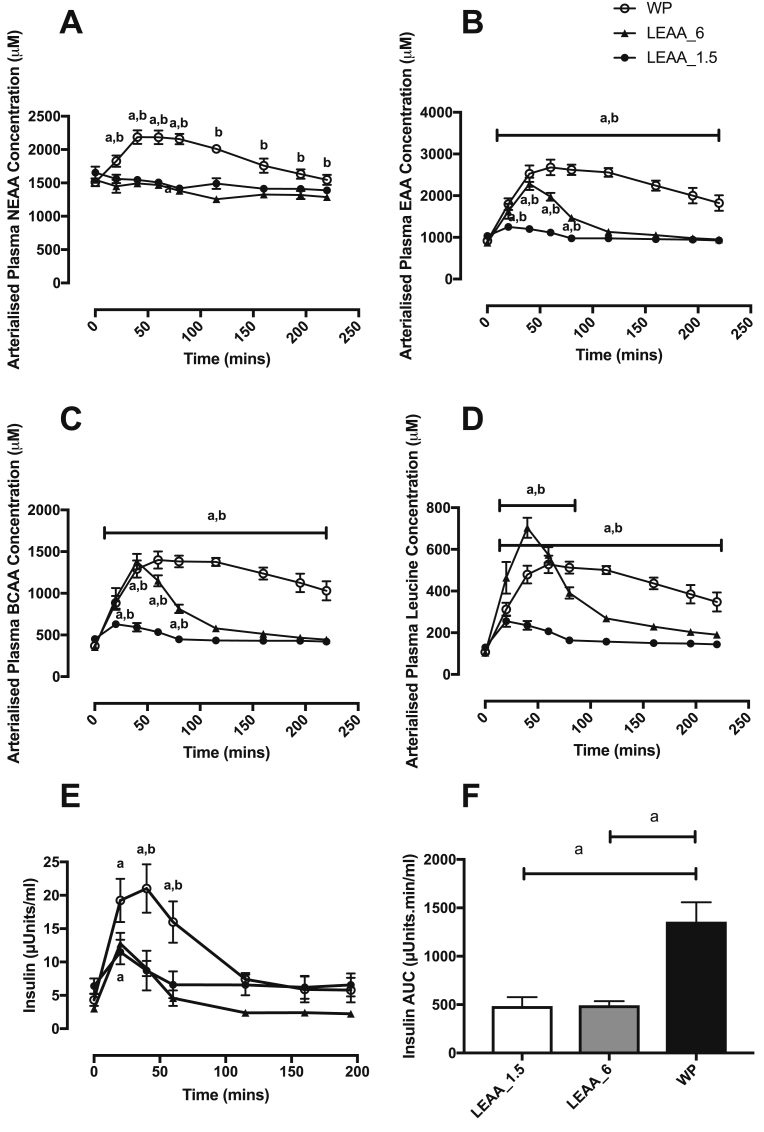
Time course effects of 1.5 g, 6 g of LEAA or 40 g of WP on plasma amino acids (AA) and insulin concentrations: nonessential AA (NEAA; A), essential AA (EAA; B), branched-chain AA (BCAA; C), leucine (D), insulin (E), and insulin AUC (F). a: significantly different vs. basal (*P* < 0.05). b: significantly different between groups (*P* < 0.05).

Plasma EAA (Fig. 2B), BCAA (Fig. 2C) and leucine (Fig. 2D) concentrations increased rapidly in both the LEAA-6 and WP groups peaking at ∼40–60 min. In the LEAA_1.5 group there was no significant increase in plasma leucine, BCAA or EAA concentrations above baseline. As expected WP showed greater plasma AA availability than both the LEAA groups with EAA, BCAA and leucine concentrations remaining significantly elevated above baseline up to 220 min, whereas concentrations in the LEAA_6 group returned to baseline within 80 min. Plasma NEAA only increased significantly in the WP group (Fig. 2A). Plasma insulin concentration increased over postabsorptive values in all groups, peaking at 11.5 ± 1.8 uU/mL, 12.8 ± 1.6 uU/mL and 21.0 ± 3.6 uU/mL, for LEAA_1.5, LEAA_6 and WP respectively (Fig. 2E). However, while plasma insulin returned to near basal concentration 40 min after both 1.5 g and 6 g LEAA intake, insulin concentrations in response to 40 g of WP were significantly elevated up to 60 min, with significantly higher insulin concentrations at 20, 40 and 60 min compared to the LEAA. The area under the curve for insulin was significantly greater in response to WP (AUC: 1358 ± 199.4 (WP) vs. 486 ± 92.1 (LEAA_1.5), 494 ± 41.4 (LEAA_6)).

### 3.2 Leg blood flow (LBF) and microvascular blood flow (MBF)

**Fig. 3:**
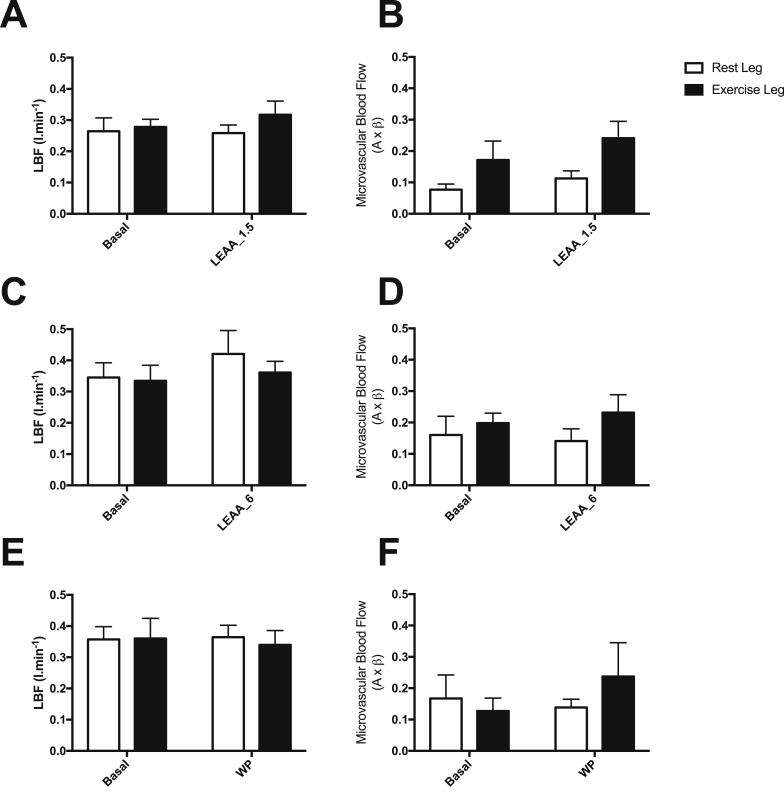
The effect of 1.5 g, 6 g of LEAA or 40 g of WP in skeletal muscle of older women on leg blood flow (LBF: A, C & E) and microvascular blood flow (MBF: B, D & F) responses in the rest and exercise legs.

Due to technical issues with the collection of these measurements, MBF and LBF data was only able to be collected for *n* = 6/group. No significant increases in MBF or LBF were evident in response to FED, or FED-EX (Fig. 3); nonetheless, it cannot be ruled out that lack of an effect may have been due to decreased numbers within each group.

### 3.3 Effects of WP and dose response LEAA on MPS

**Fig. 4:**
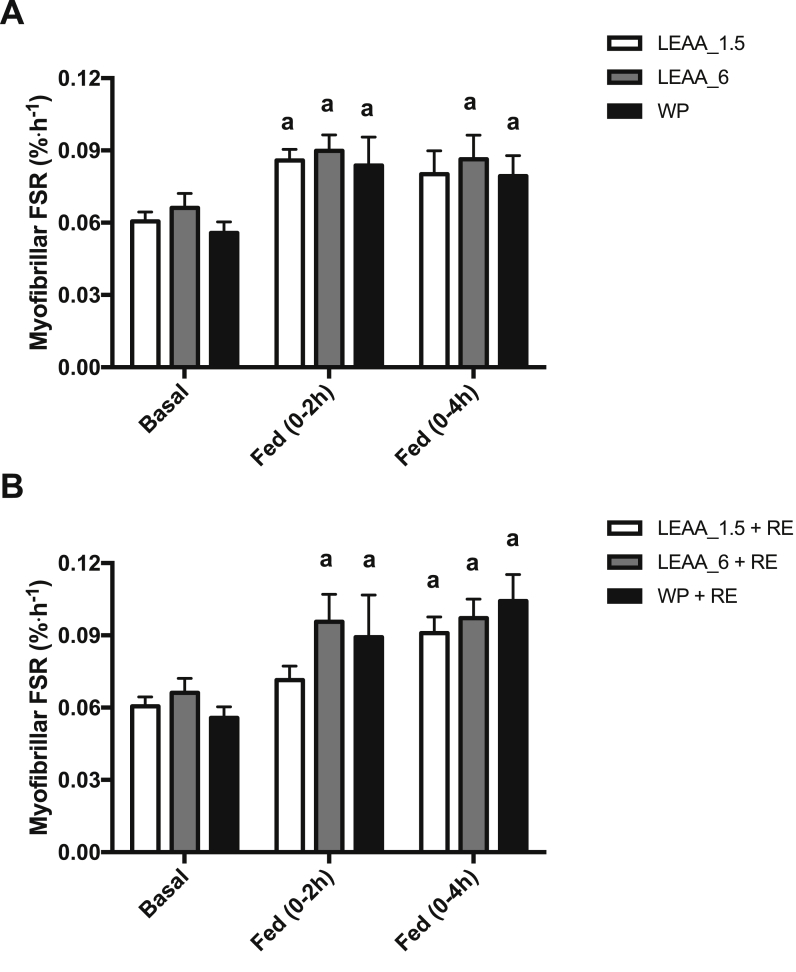
The effects of 1.5 g, 6 g of LEAA or 40 g WP on skeletal muscle myofibrillar protein synthesis in A) FED and B) FED + EX. a: significantly different vs. basal (*P* < 0.05).

In the FED leg MPS increased similarly in response to LEAA_1.5 (0.061 ± 0.003 vs. 0.086 ± 0.005%/h, *P* < 0.05), LEAA_6 (0.066 ± 0.006 vs. 0.090 ± 0.007%/h, *P* < 0.05) and WP (0.056 ± 0.004 vs. 0.084 ± 0.011%/h, *P* < 0.01) over 0–2 h, with MPS significantly greater than basal in the LEAA_6 and WP groups only over 0–4 h (Fig. 4A). However, in the FED-Ex leg, MPS significantly increased across all the groups from 0 to 4 h (LEAA-1.5, 0.061 ± 0.003 vs. 0.091 ± 0.007%/h, *P* < 0.01; LEAA-6, 0.066 ± 0.006 vs. 0.097 ± 0.007%/h, *P* < 0.01 and WP, 0.56 ± 0.004 vs. 0.104 ± 0.011%/h, *P* < 0.001; Fig. 4B). There were no between group differences in MPS at any time point.

### 3.4 Effects on muscle anabolic signalling

**Fig. 5:**
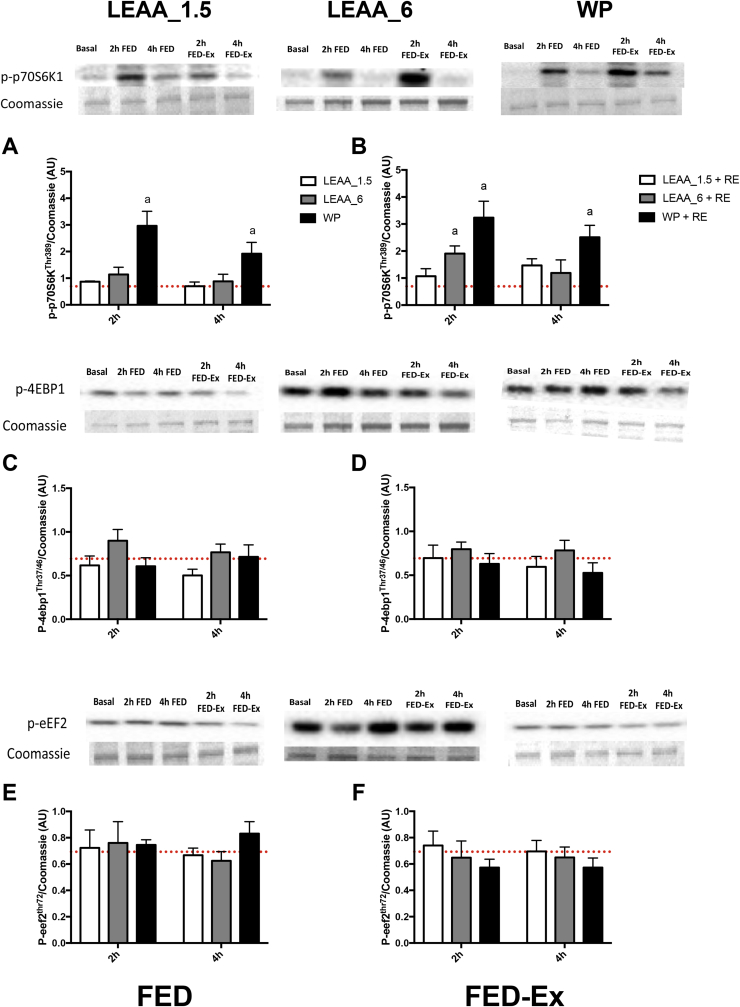
The effects of 1.5 g, 6 g of LEAA or 40 g WP on muscle signalling responses to FED and FED-EX. Responses were log transformed and normalized to the basal-fasted biopsy (represented by dotted line). a: significantly different vs. basal (*P* < 0.05).

Anabolic signalling relating to the control of MPS was assessed by measuring the mechanistic target of rapamycin complex 1 (mTORC1) translational initiation substrates; p70S6K1 and 4E-BP1, and the elongation factor eEF2. In the FED state, only p-p70S6K1 was significantly elevated in the WP group at 2 h and 4 h (Fig. 5A), however with FED-EX phosphorylation of p70S6K1 was elevated in both the LEAA_6 and WP groups at 2 h, with phosphorylation remaining elevated at 4 h in the WP group only (Fig. 5B).

## 4 Discussion

The presence of anabolic resistance in ageing has become increasingly well documented over recent years, with reports of increases in MPS being significantly dampened in older compared to younger people in response to anabolic stimuli (i.e exercise; [18], [19], nutrition; [13], [35], nutrition & exercise; [36], as well as the anti-catabolic effects of insulin [37]). Indeed, more recent evidence has suggested older individuals require greater protein intakes (vs. young) to achieve similar increases in MPS [16], [17], [38]. However, little is known of how nutritional regimens and exercise impact upon skeletal muscle anabolism in older women (who remain relatively poorly studied vs. men). In the present study we investigated, in older women, the effects of, and interaction between, the two major extrinsic drivers of muscle maintenance: nutrition and exercise. Expanding on our previous finding that a 3 g dose of LEAA stimulates MPS to a similar extent to a 20 g WP bolus [27], we report the novel findings that there does not appear to exist a bone fide dose–response in MPS to AA feeding (±exercise), in older women. Older women are an often under-represented population within nutritional research, this is despite the well-established sexual dimorphism that exists in terms of muscle protein turnover, for example MPS has been shown to be less responsive to feeding in older women compared to older men [29]. Therefore, one cannot assume that findings from males, with regards to impact on MPS during nutritional or exercise based interventions, are representative of females, hence the interest in and importance of the findings presented herein.

**Fig. 6:**
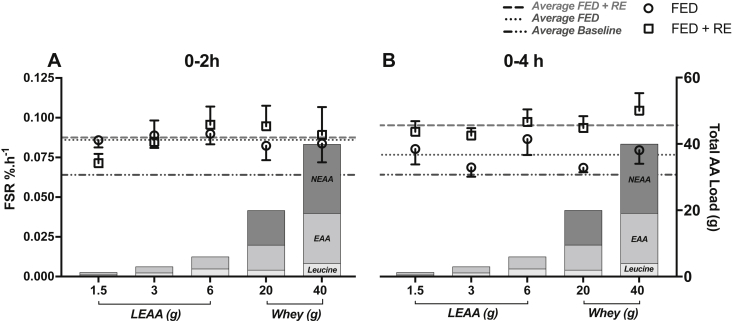
Summary dose–response relationship between MPS and AA feeding (±RE) in older women (including data from Bukhari et al. [27]). Over 0–2 h, feeding increased MPS with little enhancement with RE (A). Over the entire 4 h (B) postprandial measurement period, MPS was elevated although diminished with feeding, whilst RE sustained elevated MPS. All groups displayed similar MPS in response to feeding (±resistance exercise) suggesting only a small dose of leucine and/or EAA are required in combination with exercise to maintain elevations in MPS post nutrition, at least over the first 4 h. Left Y axis shows FSR for each feed (open circles FED, open squares FED + RE) whilst dotted lines show average FSR of all groups for baseline, FED and FED + RE. Right Y axis shows total AA load and content of leucine, EAA and NEAA.

It has been known for ∼25 y that EAA are the primary drivers behind stimulations in MPS through feeding [22], [23] – with leucine being central to this. Indeed, leucine enriched essential AA (10 g with 3.5 g Leu) prolongs MPS stimulation with RE in older men [39], with a high proportion of leucine suggested as being important for optimal stimulation of MPS by EAA in older adults [40]. Moreover, we have also previously reported that just 3 g of leucine alone stimulates MPS to near *maximal* levels (equivalent to that of a large bolus of protein) in young men [24] and also that 3 g of a novel LEAA (1.2 g of leucine) supplement led to a stimulation of MPS in older women that was equivalent to 20 g of WP [27]. Building on these previous findings, we found here that providing as little as 1.5 g of LEAA (equivalent to 0.6 g leucine and 0.9 g EAA), stimulated fed state MPS to a similar extent to that of 40 g WP, an amount suggested to maximally stimulate MPS at least in older men [30]. Nonetheless, despite 1.5 g LEAA providing identical stimulation of MPS at 0–2 h in the rested leg, this dose was unable to *sustain* significant increases in MPS over the entire 4 h postprandial measurement period, whereas MPS was still raised in both the 6 g LEAA and 40 g WP at this time. This suggests 6 g LEAA (and 40 g WP) may be *marginally* more anabolic than 1.5 g LEAA and 3 g LEAA [27] in older women (see Fig. 6 for summary), particularly when it comes to sustaining MPS and hence overall anabolism over extended periods. However, based on these findings (and others; [27]), LEAA (dosing with as little as 1.5 g) could therefore act as a supplement to every meal to ensure robust (perhaps maximal) stimulation of MPS is achieved in older populations (particularly as LEAA are less satiating than WP) where the RDA for protein is often not met [41].

It has also been proposed that the delivery profile of the AA influences the anabolic response to nutrition, with suggestions that the rapid aminoacidemia associated with large boluses of fast absorbing protein (like WP) as being key to maximizing MPS [42], [43]. However, despite there being a rapid ∼3 fold peak in plasma leucine, EAA and BCAA concentrations with 6 g of LEAA, and a maintenance of higher concentrations for longer with 40 g WP, the MPS response was similar between groups. This supports previous findings whereby bolus feeding of a fixed dose of EAA (15 g), which led to rapid plasma aminoacidemia, provided no additional anabolic effects when compared with pulse/spread feeding of the same total dose, which provided lower later peaking EAA levels [8]. These present data suggest it is the composition of the feed (e.g. quantity of leucine) rather than the dose or feeding pattern being the primary driver for anabolism.

Resistance exercise remains a pursuit by which to combat loss of muscle mass with ageing [44], even despite the fact that acute anabolic responses to exercise are blunted in older age [18], [36]. The addition of exercise to feeding (i.e. FED-EX) leads to an extension of the anabolic window beyond that of nutrition alone up to 4 h. Since this occurs in all groups, this indicates only a small dose of leucine and/or EAA are required in combination with exercise to maintain elevations in MPS post nutrition, at least over the first 4 h. What is not known from our data is whether these small doses of LEAA are sufficient to i) extend the anabolic window e.g. vs. 40 g WP, and ii) stimulate a suppression of MPB to create a net positive balance (as exercise alone is known to lead to an increase in MPB in the absence of nutrition [9]). In terms of the latter, based on previous research showing that as little an increase in circulating insulin from ∼5 to 15 uU/ml is sufficient to maximally suppress MPB [45], and the fact that in the present data even with as little as 1.5 g of LEAA insulin peaked at ∼12 uU/ml (potentially driven through leucine being a potent insulin secretagogue), one may speculate that net balance is positive in this instance. However, it must be taken into consideration that there is an ageing induced blunting in insulin mediated suppression of MPB (in older men [37]), therefore the effects of LEAA on net balance require further investigation.

The study of leg and muscle blood flow though CEUS was performed to assess whether greater doses of EAA or WP may elicit greater blood flow responses. Despite WP providing ∼3 fold greater AUC for insulin, there were no differences observed in LBF, which is in line with previous work suggesting the presence of fed-state vascular resistance in older adults [33], [46]. In contrast to our previous findings [27] there was no significant increases in MBF in any group in the FED or FED-EX legs; this difference is likely due to the technical issues with the measurements in the present study (only MBF in *n* = 6 were able to be collected per group). However, despite this, we have previously shown that the pharmacological enhancement of leg and muscle blood flow above the fed response does not enhance MPS [33], therefore any increase in leg/muscle blood flow is unlikely to have any additional significant influence on muscle protein anabolism.

Our present findings support the ever-growing body of evidence in the literature for the potent *in vivo* anabolic role of leucine [24], [27], [28], [39], [47], [48], with *in vitro* data showing that leucine provides the most robust stimulation of mTORc1 of all EAA [26]. In the present study the phosphorylation of p70S6K1 was the only anabolic target increased in the FED or FED-EX state, with 40 g WP providing the most robust and sustained increase in phosphorylation, although 6 g LEAA also increased phosphorylation at 2 h in the FED-EX state. Despite this lack of increase in anabolic signalling, there was a robust stimulation of MPS for all groups. Discordance between MPS and anabolic signalling has been previously reported [7], [45] again showing that anabolic signal measurements using standard immunoblotting approaches are not sensitive quantitative biomarkers of MPS in response to nutrition and exercise, and in this instance probably reflect between group differences in serum insulin concentrations. This, in addition to the observation of blunting of anabolic signalling pathways in older age [12], [13] may explain the present findings. Nonetheless, it is possible that peak signalling events may differ between such distinct feeding regimes such that the key points of stimulation of anabolic signalling in response to each feed may not have been captured by our biopsy timings – this is an inherent limitation of such studies.

How can such a low dose of EAA lead to such a robust stimulation of MPS and where do the EAA's required to support increased MPS derive, if not provided in the diet? The increase in MPS in the absence of exogenous EAA is not an unusual phenomenon over acute periods of measurement i.e. 90–120 min. Indeed flooding dose studies, performed more than 20 years ago, showed clearly that it was possible to stimulate MPS acutely through the provision of a single bolus of EAA (∼3 g Leu or Phe) without the addition of other EAA [22], [23], [49]. For this to occur, EAA must be derived from other pools, i.e. plasma, intracellular or protein (via whole-body or tissue protein breakdown). We and others have demonstrated, that acutely, there is clearly sufficient EAA availability to sustain short-term increases in MPS. In these studies we have shown decreases in other EAA i.e. intramuscular valine fell by 30% [22], threonine by 20% [23], and indeed upon provision of a low dose whey protein (6.25 g – equivalent to only 3 g EAA) supplement blood valine, isoleucine and total EAA concentrations drop (muscle levels were not measured), yet MPS is still stimulated [50]. Clearly these falls are the result of increased demand due to elevated MPS. Furthermore, a metabolite of leucine: β-hydroxy-β-methylbutyrate (HMB: which provides no additional nutritional value), has been shown to stimulate MPS (whilst also suppressing MPB) acutely in young males despite providing no additional EAA [24]. This acute stimulated MPS response has also been demonstrated during the provision of insulin (whereby MPB will also be significantly suppressed) in the absence of additional exogenous EAA [51]. Ergo, it is clear from the literature that in the short term, the absence of additional EAA, does not acutely limit MPS. This study was conducted acutely over 4 h, as part of a larger study investigating the dose response of MPS to EAA (enriched with leucine) as a potentially less satiating (and less calorific) alternative to consuming the larger quantities of protein suggested to be needed in older age to maximize MPS [52]. Indeed the findings from the present study support recent evidence for the positive benefits of similar low dose AA supplementation (in conjunction with exercise) in supporting anabolism in older adults [53]. However, the efficacy of LEAA supplementation with meals, for maintaining muscle mass and function chronically, remains to be fully tested. Moreover, contradictory findings for the beneficial effects of chronic leucine supplementation in ageing have been reported in the past, with 2.5 g of leucine administered alongside each main meal a day showing no improvements in muscle mass or strength in healthy older men [54]. However it should be considered that there are distinct design and methodological differences between the studies of Verhoeven [54] and Kim [53] which make comparison difficult. Firstly, the chronic leucine supplementation in the work of Verhoeven [54] was performed in healthy, non-sarcopenic males, whereas the work of Kim [53] was performed specifically in pre-defined sarcopenic older adults, where supplementation may be most beneficial. Secondly, the leucine supplementation of Verhoeven [54] was provided alongside main meals, where MPS may already be near maximal and any beneficial effect could have been negligible. Finally and most importantly, as highlighted in our main aims, the work of Verhoeven [54] was performed in a group of older males, here and in the work of Kim [53] we are specifically investigating interventions in females, where known sexual dimorphism particularly in response to nutrition are well defined [14], thereby potentially explaining why differences are observed in the outcomes of the two studies.

In summary, our present data expands upon the findings from our previous work that a leucine enriched low dose amino acid supplement provides acute robust (perhaps maximal) stimulation of MPS in older women, with our present data showing as little as 1.5 g of LEAA stimulates MPS to levels equivalent to 40 g WP (a dose known to maximally stimulate MPS in adult males) both at rest and after exercise. This supports the potential and longer term evidence [53] for efficacy of low dose LEAA supplements as strategies to support muscle mass maintenance in the ageing female population. Based on these findings, it would be prudent to investigate the efficacy in larger cohorts to see if these acute responses to low dose LEAA are translatable and reproducible over more chronic supplementation periods.

## Statement of authorship

PJA, HK, KS, PLG, JW & JL conceived and designed the study. SB, WKM & ML performed all data collection. DJW, BEP, JC, ML, MSB & DR performed the sample processing, data analyses and construction of figures. All authors contributed to the preparation and drafting of the final manuscript.

## Conflict of interest statement

Hisamine Kobayashi is an employee of Ajinomoto Co., Inc. All other authors state no conflict of interest.

## Funding sources

This work was supported by Ajinomoto Co., Inc.; the Medical Research Council [grant number MR/K00414X/1]; and Arthritis Research UK [grant number 19891]. DJ Wilkinson was a post-doctoral research fellow funded through the MRC-ARUK Centre for Musculoskeletal Ageing Research awarded to the Universities of Nottingham and Birmingham.
